# Integrated approach to malaria prevention at household level in rural communities in Uganda: experiences from a pilot project

**DOI:** 10.1186/1475-2875-12-327

**Published:** 2013-09-17

**Authors:** David Musoke, George Karani, John C Ssempebwa, Miph B Musoke

**Affiliations:** 1 Department of Disease Control and Environmental Health, Makerere University College of Health Sciences, School of Public Health, Kampala, Uganda; 2 Cardiff School of Health Sciences, Cardiff Metropolitan University, Cardiff, UK; 3 School of Sciences, Nkumba University, Entebbe, Uganda

**Keywords:** Integrated approach, Malaria, Prevention, Household level, Uganda

## Abstract

**Background:**

Malaria is a major public health challenge in sub-Saharan Africa. In Uganda, malaria is the leading cause of morbidity and mortality especially among children under five years of age. This pilot project promoted prevention of malaria at household level using an integrated approach in two rural communities in Wakiso District, Uganda. This involved advocating and implementing several strategies in a holistic manner geared towards reduction in the occurrence of malaria. The specific strategies involved can be classified as: 1) personal protection – use of insecticide-treated bed nets and insecticide sprays; 2) reducing mosquito breeding sites – draining pools of water, larviciding and clearing unnecessary vegetation around homes; and 3) reducing entry of mosquitoes into houses – installing mosquito proofing in windows, ventilators and open eaves, and closing windows and doors early in the evenings.

**Case description:**

The objectives of the project were to: carry out a baseline survey on malaria prevention; train community health workers and increase awareness among the community on the integrated approach to malaria prevention; and, establish demonstration sites using the integrated approach. A baseline survey among 376 households was conducted which generated information on the knowledge, attitudes and practices of the community in relation to malaria prevention. The project trained 25 community health workers and over 200 community members were sensitized on the integrated approach to malaria prevention. In addition, 40 demonstration households using the integrated approach were established.

**Discussion and evaluation:**

The use of multiple methods in the prevention of malaria was appreciated by the community particularly the demonstration households using the integrated approach. Initial project evaluation showed that the community had become more knowledgeable about the various malaria prevention methods that were advocated in the integrated approach. In addition, some of the methods that were not being used before project implementation, such as early closing of windows, had been adopted. The presence of mosquitoes in the demonstration households had also reduced.

**Conclusion:**

The integrated approach to malaria prevention at household level was well perceived by the project community, which could be scaled up to other areas. More rigorous studies such as randomized controlled trials are also recommended to further explore the public health impact of the integrated approach to malaria prevention.

## Background

Malaria is a major public health challenge particularly in sub-Saharan Africa. In Uganda, malaria is the leading cause of morbidity and mortality especially among children under five years of age [1,2].

Although most malaria vector control strategies in Africa have focused on the use of insecticide-treated nets (ITNs) and indoor residual spraying (IRS) [3-5], a number of other measures can be implemented at household level to significantly reduce mosquito vectors which transmit malaria. These include installing screening in windows, ventilators, and eaves to prevent entry of mosquitoes; eliminating breeding places of mosquitoes notably stagnant water; and reducing vegetation near houses where mosquitoes harbour [6,7].

Female anopheline mosquitoes, which transmit malaria to humans by biting them usually at night while in their houses, normally enter through windows, ventilators, eaves, and ceilings [8,9]. Therefore screening windows, ventilators and open eaves can prevent entry of mosquitoes into houses. In addition, closing doors and unscreened windows early in the evenings also reduces mosquito entry. This subsequently reduces chances of mosquito bites, hence potentially lowering the occurrence of malaria, where mosquito-feeding habits are indoors [10,11]. Although it has been demonstrated for many years that people could be protected from malaria by screening their homes against mosquitoes, this intervention remains virtually ignored in many communities [11]. For mosquitoes that manage to enter houses, insecticide sprays can be used to kill them. However, these are usually expensive and concerns of mosquitoes developing resistance to the insecticides have been raised [4].

Mosquitoes breed in pools of water that can be found near houses. Eliminating such sites would reduce mosquito populations that transmit malaria, due to lack of breeding sites. Draining pools of water, levelling land, construction of drains, and providing proper waste water management facilities can be carried out to eliminate mosquito breeding sites [12].

Larviciding has been used for many years as a vector control method to kill mosquito larvae where mosquitoes breed [13]. The method has been used mainly for breeding sites which cannot be drained such as those resulting from brick making in many developing countries. Larviciding has been recommended to supplement core interventions such as ITNs and IRS in the control of malaria in sub-Saharan Africa [14].

Mosquitoes are known to use vegetation as resting places [15,16], which can be seen near homes in several communities. It is from such resting places that mosquitoes approach and enter houses, commonly in the evenings and at night, from where they transmit malaria [6,17]. Consequently, maintaining vegetation near houses facilitates the presence of mosquitoes in an area because of availability of resting places [18]. Harbouring mosquitoes near houses also facilitates their entry because of the reduced distance they have to travel. Clearing unnecessary vegetation around homes can therefore reduce anopheline mosquito populations and subsequently the risk of malaria transmission.

This paper reports on a pilot project that promoted an integrated approach to malaria prevention at household level in two malaria-endemic rural communities in Wakiso District, Uganda. This involved advocating and implementing several strategies in a holistic manner geared towards reduction in mosquito populations. The specific strategies involved can be classified as: 1) personal protection – use of ITNs especially for pregnant women and children under five years of age, and insecticide sprays; 2) reducing mosquito breeding sites – draining stagnating water including filling holes and ditches with soil, and removing vessels that can potentially hold water for mosquito breeding; larviciding in large pools of water which cannot be easily eliminated such as those resulting from brick making and sand mining; and clearing unnecessary vegetation around homes; and 3) reducing entry of mosquitoes into houses – installing mosquito proofing in windows, ventilators and open eaves, and closing windows and doors early in the evenings.

The project had the following objectives:

1. To conduct a baseline survey on malaria prevention in the project area;

2. To train community health workers (CHWs) on the integrated approach to malaria prevention;

3. To increase awareness on the integrated approach to malaria prevention among the community;

4. To establish demonstration sites implementing the integrated approach to malaria prevention within the community;

5. To document key lessons learned from the project.

The project was implemented during 2011 and 2012.

## Case description

### Intervention areas

The project sites were Mayanzi zone, Kigungu, Entebbe Municipality (0.0500° N, 32.4600° E) and Lukose zone, Ssisa sub-county (0.4000° N, 32.4833° E), both in Wakiso District, Uganda. The inhabitants of the project sites were engaged in various economic and social activities such as petty trading, crop farming, animal husbandry and fishing. Malaria is the leading cause of morbidity and mortality in these areas as is the case in most parts of the country.

### Implementing partners

The project was implemented by collaboration between Makerere University School of Public Health, Uganda and Cardiff Metropolitan University, School of Health Sciences, UK.

### Project design and achievements

#### Baseline survey

A baseline survey was conducted to assess the knowledge, attitudes and practices of the community on malaria prevention and control. The survey utilized both qualitative and quantitative methods of data collection. Quantitative data was collected from 376 randomly selected households using a questionnaire and observational checklist. Whereas the questionnaire gathered information from the inhabitants on malaria prevention, the checklist assessed the environmental conditions at households that are associated with occurrence of malaria. The latter included observing the presence of mosquito breeding sites and mosquito proofing in windows, ventilators and open eaves. Qualitative data was collected from ten key informants who were mainly CHWs, local leaders and health practitioners.

The baseline survey established that the community was poor, with 81.9% having an average monthly income of less than $ 60. This was supported by key informants as stated by one of them: “*One of the main challenges in malaria control is that due to poverty, families cannot afford to buy mosquito nets or malaria medicine when sick.”*- Nurse.

Most of the participants (89.9%) were aware that malaria is transmitted through mosquito bites. Regarding malaria prevention, nearly all households (97.9%) lacked complete mosquito screening in windows and ventilators which facilitates entry of mosquitoes into houses. The participants were mainly aware of using mosquito nets including treated (29.6%) and untreated (81.7%). However, their use was noted to be low as confirmed by a key informant: *“Although some people sleep under mosquito nets, others just wait till they get malaria then they seek treatment.”* – Community leader.

Households with at least one mosquito bed net were 45.5% while only 0.5% had undergone IRS in the previous 12 months. Although there was high interest by the community in using bed nets, several challenges were established that affected their use including large family size. Indeed, 69% of the households had 4 or more members. *“Many people have large families therefore cannot afford to buy mosquito nets for all household members.”* – Village health team (VHT) member.

It was established that the government of Uganda had supported malaria control efforts in recent years such as through providing malaria medication for children to CHWs and distributing ITNs. However, these were not satisfactory. Participants who reported the availability of malaria medication with CHWs in the area were only 9.7% while 71.4% travelled more than 1 km for advice/treatment when their child had malaria. “*Although the government gave out mosquito nets a few years ago, they were very few. Only about 10% of the population received the nets which were mainly for children and pregnant women.”* – Parish chief.

The findings from the baseline survey were crucial in establishing and quantifying the knowledge, attitudes and practices of the community towards malaria prevention and control. This information was used by the project team while preparing appropriate messages and materials for community sensitization on malaria prevention. The baseline survey findings were disseminated to the community during meetings organized by the project.

### Training of community health workers

CHWs were trained on the integrated approach to malaria prevention so that they could promote these practices among the community. This involved holding training workshops in the respective communities. CHWs mainly comprised VHT members, local leaders and youth involved in health promotion. VHTs are volunteers who serve their communities and carry out health promotion and social mobilization activities. They also carry out a range of health interventions across the spectrum of health, water and sanitation, disease surveillance, and treatment of common illnesses. A total of 25 CHWs in the two villages were trained. During the training, CHWs were informed about their responsibility of promoting the integrated approach to malaria prevention in their respective communities even beyond the project period. This was a key sustainability strategy of the project.

### Community sensitization

**Figure 1:**
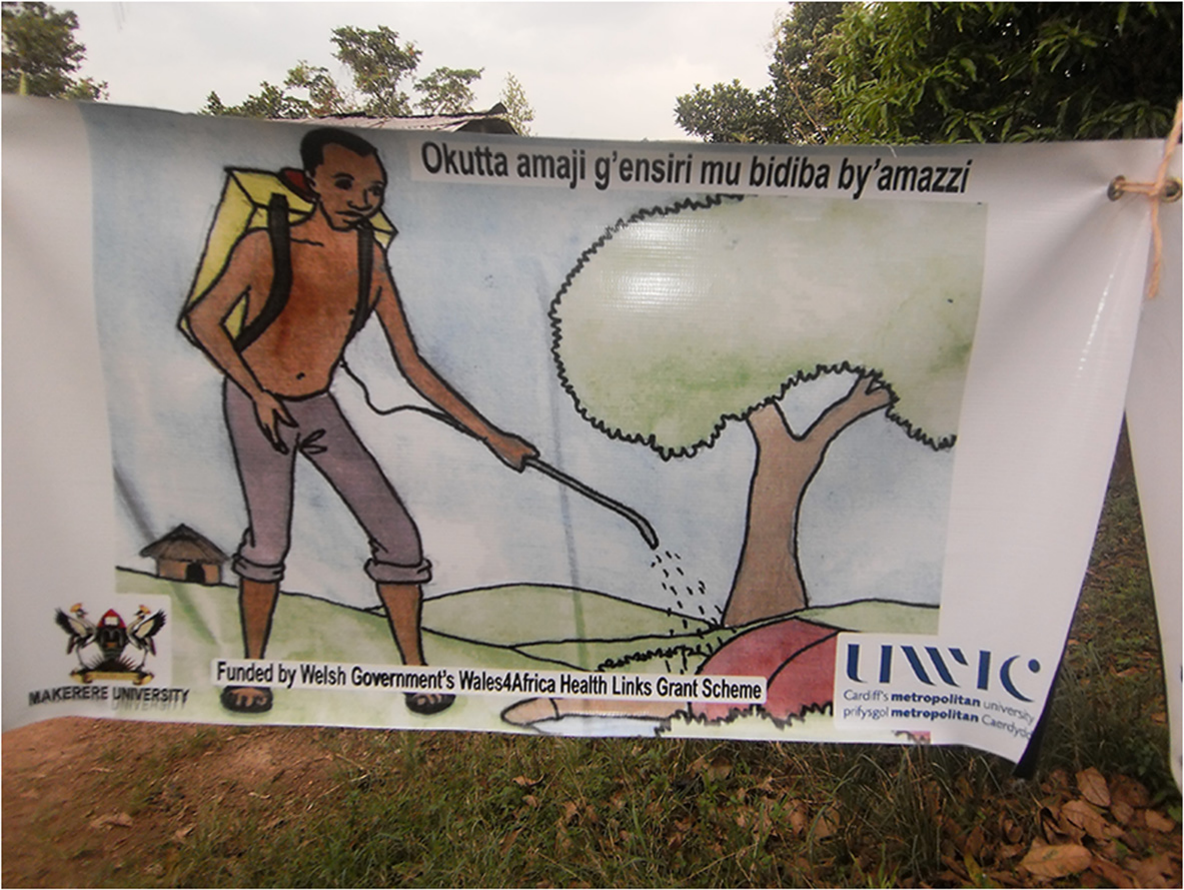
One of the posters used for training community health workers and sensitization.

**Figure 2:**
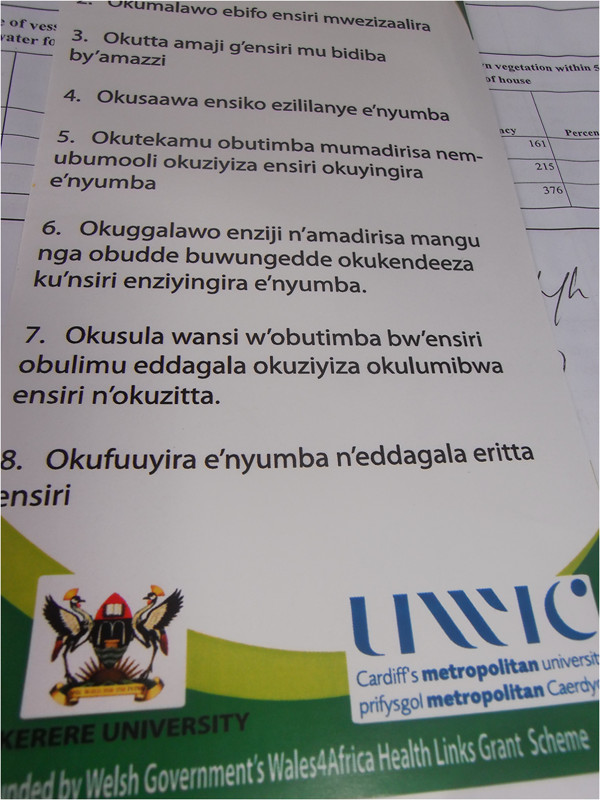
Part of the flier used for training community health workers and sensitization.

The community in the project sites was sensitized on the integrated approach to malaria prevention. This involved holding sensitization sessions in the communities. Over 200 community members in the two project areas were sensitized by the project. Both training of CHWs and community sensitization involved use of appropriate information, education and communication (IEC) materials such as posters and fliers, in addition to health talks (Figures 1 and 2). Eight posters were developed for the training, each containing one malaria prevention strategy being advocated in the integrated approach. This included an illustrative presentation and name of the strategy. This design was meant to capture the attention of the community as well as the messages being understood by those who could not read. The flier contained all the eight malaria prevention methods being advocated in the integrated approach. Several copies of the fliers were distributed to the community after the training so as to give them out to others who did not attend. These materials were translated into the local language (*Luganda*) so as to facilitate learning among the population.

During the community sensitization sessions, emphasis was on children under five years of age and pregnant women as the groups most affected by malaria. This helped to target Millennium Development Goals (MDGs) 4 (reducing child mortality) and 5 (improving maternal health) in addition to MDG 6 (involving combating malaria) which was the main focus of the project.

### Establishing demonstration sites

Model households were set up in the project community implementing the integrated approach to malaria prevention. These were used as demonstration sites in the project areas. A total of 40 demonstration households (20 in each project site) were established. These were selected by the community leaders following the guidelines provided by the project. The project required that priority be given to households that had children under five years of age and/or a pregnant woman, and the demonstration households needed to be well distributed in the community. It was important that the demonstration households were evenly distributed in the community so that majority of the population could have access to at least one such household.

**Figure 3:**
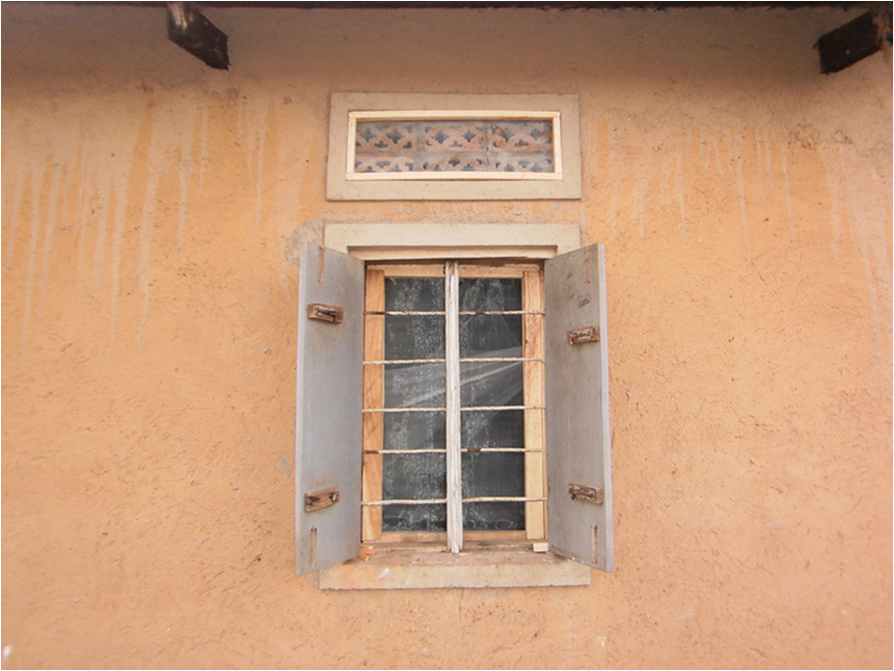
A window and ventilator on one of the demonstration houses with complete mosquito proofing.

**Figure 4:**
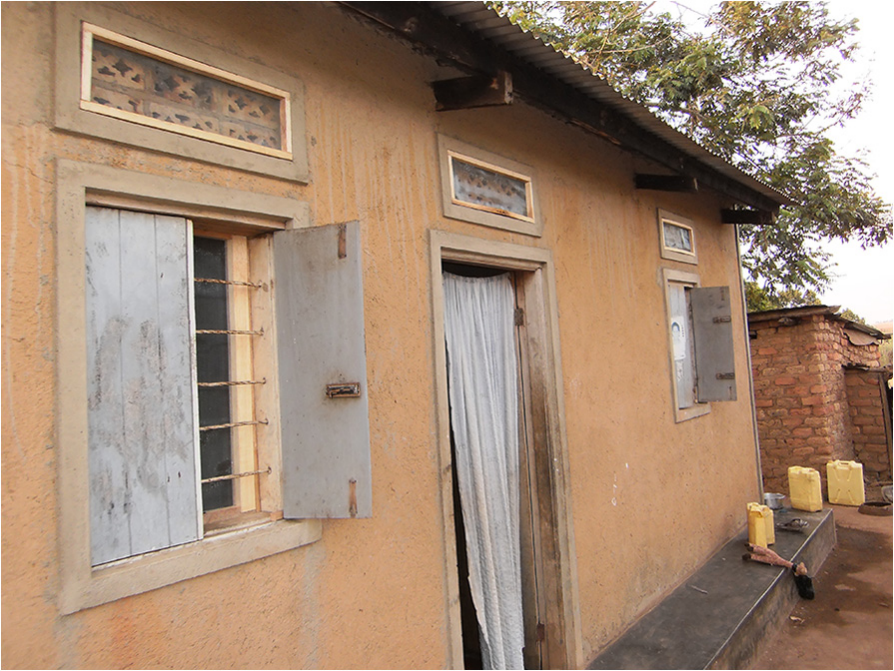
One of the demonstration houses with complete mosquito proofing in windows and ventilators.

As part of the integrated approach, the project installed complete mosquito proofing in all windows and ventilators of the demonstration houses to prevent mosquito entry (Figures 3 and 4). This involved procurement of the necessary materials including rolls of mosquito proofing, small pieces of timber and nails. The installation of mosquito proofing was done by experienced carpenters. The project also provided long-lasting insecticide nets (LLINs) for use by members of the demonstration households. The number of nets received per household depended on the number of household members and the available functional nets at the time. Households received between two and six LLINs. These interventions were carried out after educating the beneficiaries on the importance of using the methods in the prevention of malaria. These demonstration households were important in promoting the integrated approach among the community. The interventions were not only beneficial to the members of the demonstration households but also the entire community who appreciated the integrated approach, which they had been taught during the project training. Indeed, several community members on seeing the demonstration households expressed interest to the project team in having the interventions also implemented in their houses. In addition, villages neighbouring those involved in the project requested the project team to extend the interventions to their areas. However, this was not possible mainly due to limited resources. It was the responsibility of members of respective demonstration households to implement the other strategies in the integrated approach such as closing doors early in the evenings to prevent mosquito entry and removal of mosquito breeding sites. These demonstration households were expected to continue to be used to promote the integrated approach in the community even beyond the project period, including for future long-term evaluation activities.

## Ethical considerations

Approval to implement the project was obtained from the Makerere University School of Public Health Higher Degrees, Research and Ethics Committee. The project was also registered at the Uganda National Council for Science and Technology. The local leaders of the villages were duly informed about the project. Written informed consent was obtained from the baseline survey participants and heads of demonstration households before taking part in the project.

## Discussion and evaluation

The baseline survey findings showed that the community was poor, strategies targeting prevention of malaria were few, and several challenges in treatment of malaria existed. Such surveys done before implementation of projects are key in establishing the actual situation in the community before designing interventions [19]. The survey established a low use of core WHO malaria prevention methods of ITNs and IRS. This, therefore, necessitated the implementation of this project in these areas, which advocated for use of multiple methods at households in a holistic manner. Baseline surveys also provide data that can be used during monitoring and evaluation of interventions [20] as was the case in this project.

CHWs are known to greatly increase access to health services especially among rural and hard-to-reach communities [21]. As people involved in health service delivery, they can be utilized to carry out health promotion [22]. The incorporation of promoting the integrated approach in their work was well received as they were already involved in malaria control work. However, adding more responsibilities to CHWs needs to be carefully considered so as not to lead them to exhaustion as has been observed among social workers [23].

The use of local languages in project activities, including sensitization, is very important in rural communities because majority of the inhabitants do not understand English, the country’s official language. Although malaria affects all categories of people, it is also important to recognise the high-risk groups of children and pregnant women as recommended by WHO [24]. This ensures that with limited resources, priority is given to those at most risk and hence need such as children under five years of age using the available ITNs in households.

Use of demonstrations has been shown to promote community-based health programmes including sustainability of interventions [25]. The demonstration households were well distributed in the areas so as to ensure a wide geographical coverage. This enabled increased access of community members to these households. The project interventions among these households of provision of LLINs and screening of houses against mosquitoes were beneficial given the financial constraints in rural communities. Nevertheless, it was important that the households had responsibilities to accomplish on their own, such as early closing of doors and removal of mosquito breeding sites. Indeed, the implementation of such simple measures at the households by the project team was impractical and would reduce community participation. In addition, carrying out all activities for households would not have been sustainable.

Initial project evaluation was carried out through interviews with community members including demonstration households. Members of the demonstration households reported fewer mosquitoes in their houses following the project’s interventions. It was also established that after sensitization, the community was more knowledgeable about the various malaria prevention methods that were advocated in the integrated approach. Some of the interventions that were not being used before project implementation were being practiced in the area. This included early closing of doors and windows, and removal of mosquito breeding sites. However, other methods, such as larviciding, were not being used because of the high costs of commercial larvicides involved. Although some methods in the integrated approach were not implemented, the ones that were being used on their own play a significant role in malaria prevention. A combination of multiple malaria prevention strategies has been shown to have greater impact than single methods in some studies [26,27].

### Project challenges

There were only four official VHT members in each village that constituted the trained CHWs yet they had to work in relatively large geographical areas. The other trained CHWs, such as local leaders, are normally not involved in health promotion work as much as VHTs. This reduced the impact such human resource could have had to improve health in their communities. In addition, the VHTs were found with existing challenges such as minimal training and low motivation.

The project was implemented in only two villages. Therefore, a greater impact could have been realised if resources permitted the involvement of more communities in the project. Indeed, neighbouring communities to the project sites showed interest in project activities. Only 40 demonstration households were established, yet more households wanted to benefit from the interventions that were implemented.

The project was implemented in a short period of time. It was therefore not possible to measure the long term impact of the interventions among the community such as reduced occurrence of malaria. However, an impact evaluation is to be conducted over two years after implementation of the project. This evaluation will provide more information on the public health impact of using the integrated approach to malaria prevention at household level.

### Key lessons learnt

From this pilot project, it was established that a strong team of community mobilizers is imperative for the success of community programmes. These mobilizers were crucial in mobilizing the community during various project activities, such as meetings and community sensitization sessions.

Obtaining the goodwill of local leaders and officials from the health departments before project implementation was of paramount importance to the success of the project. These personnel were not only actively participating in project activities but also mobilizing their community members for various interventions.

## Conclusion

The integrated approach to malaria prevention at household level was well perceived by the project community, which improved their knowledge and practices on malaria prevention. With the success of this pilot project, a wider population could benefit from similar interventions. More rigorous studies, such as randomized controlled trials are also recommended to further explore the public health impact of the integrated approach to malaria prevention.

## Abbreviations

CHWs: Community health workers; IEC: Information education and communication; ITNs: Insecticide-treated nets; LLIN: Long-lasting insecticide net; MDG: Millennium development goal; IRS: Indoor residual spraying; VHT: Village health team; WHO: World Health Organization.

## Competing interests

The authors declare that they have no competing interests.

## Authors’ contributions

The project principal investigators (DM and GK) conceived and implemented the project, and did the main writing of the manuscript. JCS and MBM were involved in project implementation and offered critical comments in the reviewing and writing of the manuscript. All authors read and approved the final version of the manuscript.
